# Understanding the use of spheroids and pellets in the chondrogenic differentiation of human stem cells

**DOI:** 10.1016/j.ocarto.2026.100779

**Published:** 2026-03-14

**Authors:** Donna-Madina A.J. Sangen, Stefan Giselbrecht, Martijn van Griensven, Steven Vermeulen, Elizabeth R. Balmayor

**Affiliations:** a Experimental Orthopaedics and Trauma Surgery, RWTH Aachen University Hospital, Aachen, Germany; b Department cBITE, MERLN Institute for Technology-Inspired Regenerative Medicine, Maastricht University, Maastricht, The Netherlands; c Department CTR, MERLN Institute for Technology-Inspired Regenerative Medicine, Maastricht University, Maastricht, The Netherlands; d FIERCELab, Biomedical Research Institute, Hasselt University, Hasselt, Belgium

**Keywords:** Pellets, Spheroids, Microwell culture, Stem cells, Chondrogenesis, Hypertrophy

## Abstract

**Objectives:**

The limited regenerative capacity of cartilage tissue and the high morbidity associated with injuries and diseases have driven the search for innovative regenerative medicine strategies. The objective of the study was to compare the chondrogenic differentiation of human MSCs in conventional pellet cultures to that of spheroids generated using an innovative microwell system.

**Design:**

Human bone marrow mesenchymal stem cells (hBMSCs) were isolated and cultured in either pellet or microwell systems. Upon induction of chondrogenesis, gene expression and extracellular matrix deposition were analysed.

**Results:**

We found that chondrogenic pellets outperformed spheroids based on the expression levels of chondrogenic markers, such as *SOX9*, *COL II*, *COLIXa2*, *COLXIa2*, *ACAN*, *VCAN*, and the trio *SOX5*, *SOX6*, and *SOX9*. However, hypertrophic markers, such as *COL X*, *RUNX2*, *COLI*, and *MMP13,* were higher in chondrogenic pellets. *DCN* and *BGN* expression, along with increased *COMP* expression in the microwell spheroids, may reflect a role in matrix stabilisation and network organisation rather than chondrogenic differentiation. Histological analysis demonstrated a richer extracellular matrix deposition in the chondrogenic pellet culture, while the spheroids exhibited less calcification.

**Conclusions:**

This study demonstrates the complexities of MSC's chondrogenesis across different aggregate dimensions in balancing chondrogenesis and hypertrophy. Overall, these findings indicate that the culture system choice should reflect specific biological and translational aims, with each system offering complementary strengths.

## 1 Introduction

Articular cartilage has limited regeneration capabilities, contributing to the initiation of diseases like osteoarthritis. The lack of cartilage self-healing capacity and the considerable morbidity caused by cartilage injuries and diseases have prompted novel research on regenerative medicine approaches. For this, developing robust in vitro models is essential for understanding chondrogenic-associated mechanisms and for developing clinical models. A challenge remains in recapitulating an in vitro environment suitable for chondrocyte investigations. While chondrocytes have been utilised for cartilage engineering, their limited availability [1], along with their tendency to lose chondrocyte phenotype [2], present a significant challenge. To potentially overcome this shortage, cartilage engineers have long turned to Mesenchymal Stem Cells (MSCs) in search of an advantageous cell source [3]. Using stem cells, however, implies that culture and microenvironmental conditions need to be tightly controlled to generate and maintain the right phenotype. Three-Dimensional (3D) culture systems, such as pellets, spheroids, or the use of microspheres, offer significant advantages over traditional monolayer cultures. These systems better replicate the structural and functional complexity of native tissues [4]. Through dynamic cell-cell and cell-matrix interactions, they can support enhanced differentiation and Extracellular Matrix (ECM) formation [5]. Such 3D models reveal detailed insights into cell behaviour, differentiation, and tissue formation, making them valuable tools in regenerative medicine, disease modelling, drug testing, and personalised medicine [6].

Among the 3D culture methods available, pellet culture systems have been widely used and are considered the standard methodology to support MSC chondrogenesis [5,7]. However, pellet culture systems present important drawbacks. There is spatial heterogeneity in the differentiation of cells; cells in the outer layer tend to undergo more efficient chondrogenic differentiation, while cells in the core are often undifferentiated or become necrotic [3]. In addition, MSCs tend to acquire hypertrophic properties during chondrogenic differentiation, signalling the terminal stage of chondrocyte differentiation into bone, which is unfavourable for cartilage engineering [8]. As a result, the development of more advanced 3D culture systems for cartilage engineering has been extensively studied. Microwell culture plates have been used to develop spheroids and embryoid models and have been shown to be very advantageous in bone [9,10] and organoids research targeting different tissues and organ systems [7]. Following similar reasoning, we propose here the use of microwell technology to develop stem cell spheroids for chondrogenesis research. We hypothesise that the microwell culture's unique environment may profoundly influence different pathways of preventing hypertrophy, a critical concern in cartilage engineering. The smaller diameter of the spheroids formed in the microwell system could minimise diffusion gradients for metabolites, growth factors, and gases, creating a more controlled environment for differentiation and reducing the heterogeneity often observed in MSCs differentiation within traditional pellet cultures [11]. Furthermore, microwell culture models facilitate high-throughput and scalable production of MSCs aggregates. The ability to produce hundreds of uniform aggregates simultaneously, in a controlled manner, is a significant advantage over conventional methods. By reducing well-to-well and in-well variability, microwells provide a reliable platform for consistent spheroid formation [12].

To test our hypothesis, in this study, we performed a comparative analysis of MSCs chondrogenesis between pellet and microwell culture systems by means of gene expression and histological evaluation. Particular emphasis was placed on the evaluation of chondrogenic potential and hypertrophic tendencies of MSCs cultured in both systems. Such a comparison has not been reported in the literature to date. Our findings aim to offer insights that could advance the development of more effective strategies for cartilage regeneration.

## 2 Materials and methods

### 2.1 hBMSCs Harvesting, isolation, and expansion

Bone marrow samples were used to isolate the human Bone Marrow Mesenchymal Stem Cells (hBMSCs) used in this study. Cells were cultured in monolayers at 37 °C and 5% CO_2_ by using an expansion cell culture medium (i.e., Minimum Essential Medium-α GlutaMAX (Gibco, Waltham, MA, USA), supplemented with 10% Fetal Bovine Serum (FBS) (Sigma-Aldrich, Saint Louis, MO, USA) and 1% Pen/Strep (Thermo Fisher, Waltham, MA, USA). Ascorbic acid (1%) (Sigma-Aldrich, Saint Louis, MO, USA) was supplemented fresh during medium changes. The cells received fresh medium twice a week. Passage 5 cells were used for pellet and spheroid generation.

### 2.2 Optimisation of seeding densities

To optimise the initial hBMSCs seeding densities for both pellet and microwell culture systems, a range of cell densities was tested. For pellet cultures, cell numbers ranging from 5 × 10^4^ to 1 × 10^6^ cells/cm^2^ were evaluated. Similarly, for microwell cultures, seeding densities from 5 × 10^4^ to 8 × 10^5^ cells/cm^2^ were assessed. Brightfield microscopy evaluation was performed after 24 h to determine the optimal density for each culture system. Density optimisation was based on parameters such as success of pellet and spheroids formation, pellet and spheroids sizes and shape, lack of multiple spheroids per microwell, among others. The optimised densities were used for all subsequent experiments, i.e., 10^6^ cells/cm^2^ for pellet cultures and 4 × 10^5^ cells/cm^2^ for microwell cultures.

### 2.3 Pellet culture system

hBMSCs were seeded at 10^6^ cells/cm^2^ in a V-bottom 96-well plate (Greiner Bio-One, Kremsmünster, Austria) and centrifuged at 500*g* for 5 min to facilitate pellet formation. Cells were cultured in chondrogenic medium, i.e., 1% Insulin-Transferrin-Selenium (Corning, Manassas, VA, USA), 1% Pen/Strep (Thermo Fisher, Waltham, MA, USA), 40 μg/mL proline (Sigma-Aldrich, Saint Louis, MO, USA), 10 ng/mL Transforming Growth Factor-β1 (PeproTech, Canbury, NJ, USA), 100 nM dexamethasone (Sigma-Aldrich, Saint Louis, MO, USA), and 1 μM ascorbic acid (Sigma-Aldrich, Saint Louis, MO, USA) in Dulbecco’s Modified Eagle Medium with 4.5 g/L D-glucose, 110 mg/L sodium pyruvate (Thermo Fisher, Waltham, MA, USA). Control medium consisted of 10% FBS (Sigma-Aldrich, Saint Louis, MO, USA), 1% Pen/Strep (Thermo Fisher, Waltham, MA, USA), 1% ascorbic acid (Sigma-Aldrich, Saint Louis, MO, USA) in DMEM with 4.5 g/L D-glucose and 110 mg/L sodium pyruvate (Thermo Fisher, Waltham, MA, USA). Cultures were maintained at 37 °C and 5% CO_2_, with medium refreshed every two days. Cultures were maintained for 28 days, upon which samples were collected for further analysis.

### 2.4 Microwell culture system

**Fig. 1:**
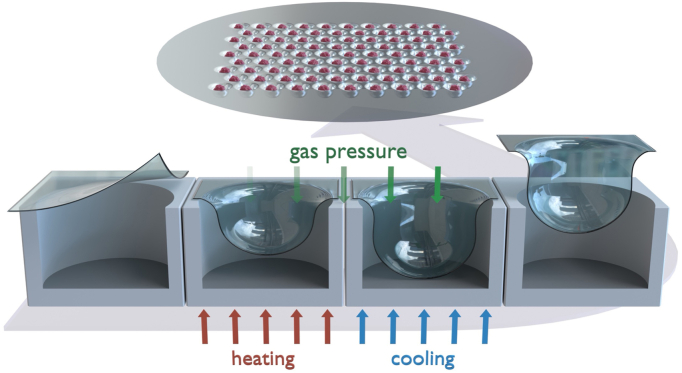
Schematic representation of the microthermoforming process for microwell fabrication. A polycarbonate film was gas pressurised and stretched into the mould's microcavities, while heated and cooled down, forming 289 hexagonally arranged blind-hole cavities with a diameter of 500 μm and 300 μm deep.

Microwells (289 wells per unit) were fabricated as circular-cylindrical dead-end holes (500 μm diameter, 300 μm depth) on a 50 μm polycarbonate film by gas-assisted microthermoforming, as described earlier [13] (Fig. 1). Microwells are available through 300MICRONS GmbH (Karlsruhe, Germany). Before cell seeding, microwells were embedded in a 24-well plate (Thermo Fisher, Waltham, MA, USA), secured with an O-ring being placed on top (ERIKS, Alkmaar, the Netherlands), sterilised with ethanol 70%, treated with Pluronics F108 (Sigma-Aldrich, Saint Louis, MO, USA) 1% coating to inhibit cell attachment, and washed three times with PBS (Thermo Fisher, Waltham, MA, USA). One microwell unit was placed in each well of a 24-well plate. hBMSCs were seeded at 4.10^5^ cells/cm^2^ per microwell unit. Cells were cultured in the chondrogenic medium as described above for a period of 28 days. Cell culture medium was refreshed every two days. Subsequent evaluations were performed as described hereafter.

### 2.5 Total RNA extraction and reverse transcriptase-polymerase chain reaction

To assess differences in gene expression between pellets and spheroids, we performed qPCR upon RNA extraction. Pellets and spheroids were lysed using TRIzol reagent (Thermo Fisher, Waltham, MA, USA), and RNA was extracted using RNeasy Mini Kit (Qiagen, Hilden, Germany), including a DNase treatment to remove genomic DNA contamination. RNA concentration and purity were quantified by spectrophotometry with a Biodrop μLite+ (Biochrom, Holliston, MA, USA). cDNA was synthesised using iScript cDNA kit (BioRad, Hercules, CA, USA) on a PeqSTAR X thermocycler (Avantor, Radnor Township, PA, USA), followed by qPCR in a CFX96 thermocycler (BioRad, Hercules, CA, USA) with iScript SYBR green kit (Bio-Rad, Hercules, CA, USA). The thermal cycling consisted of a 2-step cycling protocol for 40 cycles, followed by a melt curve analysis. Ct-values >35 were excluded. qPCRs were run in two technical replicates to ensure data reproducibility. Data was processed as relative expression (2ˆ(-ΔCt)) where ΔCt was calculated as Ct _gene of interest_ – Ct _geometric mean (reference genes)_ with respect to the corresponding donor. Relative expression values (2ˆ(-ΔCt)) from independent runs were then averaged for downstream analysis. Data treatment was performed independently for each donor.

Gene expression data were normalised using the geometric mean of three reference genes: Glyceradehyde 3-Phosphate Dehydrogenase (*GAPDH*), Cleavage and Polyadenylation Specificity Factor 6 (*CPSF6*), and TATA-Binding Protein (*TBP*). Reference gene stability was assessed using the geNORM algorithm implemented in qBASE+ (Biogazelle, Zwijnaarde, Belgium) **(**Supplementary Table 1**)**. Two independent sets of technical replicates were analysed separately. The second set showed reduced reference gene stability, whereas the first set exhibited great stability (M-values <1). As the same biological samples and reference genes were analysed in both runs, these differences were attributed to run-to-run technical variation rather than biological effects. To account for this variability, each run was normalised independently before averaging relative expression values.

### 2.6 Histology and immunohistochemistry

To evaluate phenotypic differences between pellets and spheroid cultures, pellet and spheroid aggregates were harvested at 28 days of culture, fixed in 4% formaldehyde (Sigma-Aldrich, Saint Louis, MO, USA), and embedded in cryocompound (ImmunoLogic, Amsterdam, the Netherlands) to preserve tissue morphology for cryosectioning. Cryosections (10 μm) were stained for alcian blue (targeting glycosaminoglycans) (Sigma-Aldrich, Saint Louis, MO, USA), safranin O (targeting proteoglycans) (Sigma-Aldrich, Saint Louis, MO, USA), and alizarin red (targeting calcium deposits) (VWR Chemicals, Lutterworth, UK). For immunohistochemistry (IHC), samples were blocked with 10% Bovine Serum Albumin, incubated with primary antibodies (rabbit anti-collagen II and mouse anti-collagen X, Abcam, Cambridge, UK, 1:200) overnight, followed by secondary antibodies (anti-mouse 647 and anti-rabbit 488, Abcam, Cambridge, UK, 1:200) and DAPI nuclear stain (Sigma-Aldrich, Saint Louis, MO, USA, 1:500). Slides were mounted in Diamont antifade (Thermo Fisher, Waltham, MA, USA). Stained sections were visualized using a Ti-E slide scanner (Nikon, Tokyo, Japan) for IHC and a Ti–S/L100 microscope (Nikon, Tokyo, Japan) for histological imaging. Images were processed, and semi-quantification was performed using ImageJ software (US National Institutes of Health, Bethesda, MD, USA). Semi-quantitative results are reported as the percentage of positively stained area relative to the total area.

### 2.7 Statistical analysis

A one-way ANOVA was conducted to compare differences between the experimental conditions using GraphPad Prism (version 10.4.1, GraphPad Software, San Diego, CA, USA). A p-value <0.05 was considered statistically significant. The software recommendations for statistical analysis were followed, and the statistical significance was noted as per GraphPad Prism style.

## 3 Results

### 3.1 Seeding density optimisation of pellets and microwell-formed spheroids

**Fig. 2:**
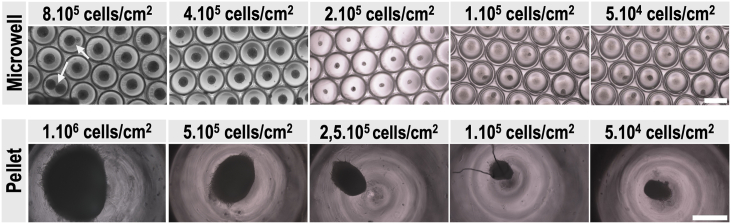
Microscopic images of pellets and spheroids formed by means of different initial hBMSCs seeding densities. Images taken at 24 h after cell seeding. The scalebar represents 500 μM. **(Top)** Microwell culture system. **(Bottom)** Pellet culture system.

Optimisation experiments were performed to determine the optimal cell densities for both pellet and spheroid cultures (Fig. 2). For pellet cultures, a seeding density of 1 × 10^6^ cells/cm^2^ was chosen, as it consistently produced robust and stable cell pellets with the largest pellet size. In the microwell system, the seeding density influenced both the formation and the number of spheroids per well. At the highest seeding density tested (8 × 10^5^ cells/cm^2^), overcrowding occurred, resulting in multiple spheroids forming within a single microwell (indicated with white arrow in Fig. 2). Lower seeding densities (≤2 × 10^5^ cells/cm^2^) produced too few spheroids to consistently populate the microwells. A density of 4 × 10^5^ cells/cm^2^ was selected as optimal; it enabled consistent formation of a single spheroid per microwell. These seeding densities were chosen to ensure that each system was studied under its most optimal culture conditions, enabling functional comparisons between pellet and microwell spheroid systems.

**Fig. 3:**
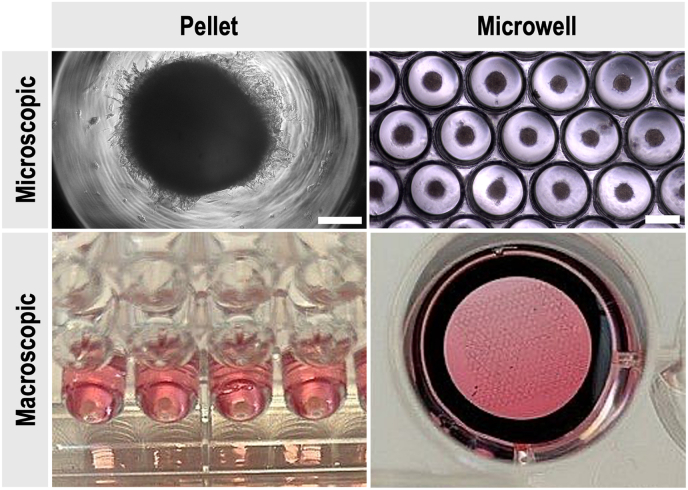
Microscopic and Macroscopic view of chondrogenic pellets and microwell-formed spheroids at 28 days of in vitro culture. **(Top)** Brightfield microscopy. The scalebar represents 300 μM. **(Bottom)** Macroscopic images of pellet and microwell culture set-up. Pellets were formed in a 96 V-shaped bottom-well plate. The microfabricated microwells were fitted in a 24-well plate and secured with an O-ring.

To further characterise both culture systems, the dimensions of spheroids and pellets were compared (Fig. 3). Spheroids were only observable microscopically, with a diameter of approximately 100 μM, whereas pellets reached sizes of up to 1500 μM in diameter. Although the cell numbers were optimised for each model, this comparison highlights intrinsic differences between the two culture approaches, such as structural compactness and scale, which may inform method selection for downstream applications.

The use of microwells ensured homogeneous distribution of cells during the initial seeding event. Individual microwells successfully divided the cell-suspension into equally sized aggregates. In contrast, seeding into a single large well resulted in the formation of a single, large pellet. This occurs even if equal cell numbers would have been chosen for both culture methods, thereby creating a clear and well-defined difference in spheroid vs. pellet dimensions. A controlled size is essential to enable the investigation of size-dependent effects on cell phenotype.

### 3.2 Gene expression in spheroids compared to pellet cultures

**Fig. 4:**
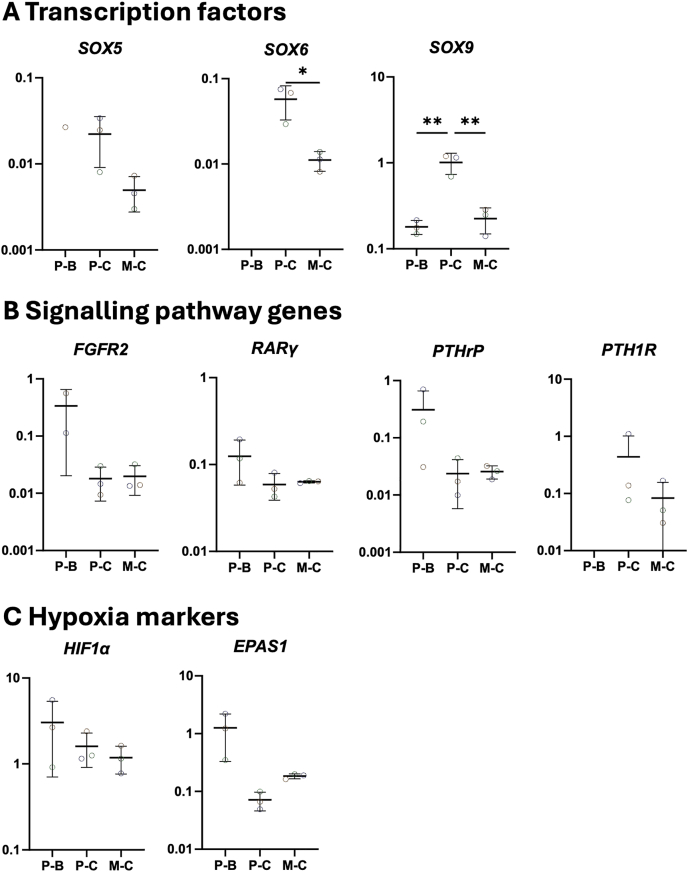
Normalised gene expression of regulation and signalling genes in pellet and spheroid cultures at 28 days of in vitro culture **A.** Transcription factors. **B** Signalling pathway genes. **C** Hypoxia markers. Dot plots represent the 2ˆ(-ΔCt). Biological replicates are n = 3. Each dot represents one donor. Abbreviations in graphs: P–B (Basic Pellet), P–C (Chondrogenic Pellet), and M – C (Chondrogenic Microwell). ∗p < 0.05; ∗∗p < 0.01; ∗∗∗p < 0.001.

To evaluate the differences in chondrogenic potential between hBMSC spheroids generated in microwell and pellet cultures, we analysed the expression of key chondrogenic, hypertrophic, and osteogenic markers. The transcription factors SRY-box Transcription Factor (*SOX*) 5, 6, and 9, critical for chondrogenesis, exhibited higher expression in chondrogenic pellets compared with spheroid cultures (Fig. 4A). *SOX9* expression was highest in pellet cultures (mean ± SD: 1.01 ± 0.23) and was significantly increased compared to both the basic pellet (0.18 ± 0.03; p < 0.01) and spheroids (0.22 ± 0.06; p < 0.01). Similarly, *SOX6* expression was significantly greater in pellet cultures (0.06 ± 0.02; p < 0.05) compared with spheroids (0.01 ± 0.002). *SOX5* expression followed a comparable trend, with higher levels in pellets (0.02 ± 0.01) relative to spheroids (0.005 ± 0.002). However, this trend was not consistent across other signalling pathways associated with chondrogenesis. For instance, no significant differences were observed in the expression of Fibroblast Growth Factor Receptor 2 (*FGFR2*), Retinoic Acid Receptor Gamma (*RARγ*), or Parathyroid Hormone-Related Peptide (*PTHrP*) between the two culture systems. Interestingly, Parathyroid Hormone-Related Peptide Receptor (*PTH1R*) did not show any expression in the basic pellets, while there was a 5.5-fold increase in the *PTH1R* expression between the chondrogenic pellet (0.44 ± 0.47) and the spheroids (0.08 ± 0.06) (Fig. 4B). Additionally, the hypoxia marker Hypoxia Inducible Factor 1-Alfa (*HIF1α*) was lowest expressed in the spheroid cultures (1.18 ± 0.35) compared to the basic pellet (3.04 ± 1.90) and chondrogenic pellet (1.60 ± 0.56), suggesting reduced hypoxic conditions. Conversely, Endothelial PAS Domain-Containing Protein 1 (*EPAS1*), also known as *HIF2α*, levels were lowest in the chondrogenic pellet cultures (0.07 ± 0.02), corresponding to an 18-fold decrease of relative expression compared with the chondrogenic pellets (1.26 ± 0.76) and a 2.5-fold decrease compared with spheroids (0.18 ± 0.01) (Fig. 4C).

**Fig. 5:**
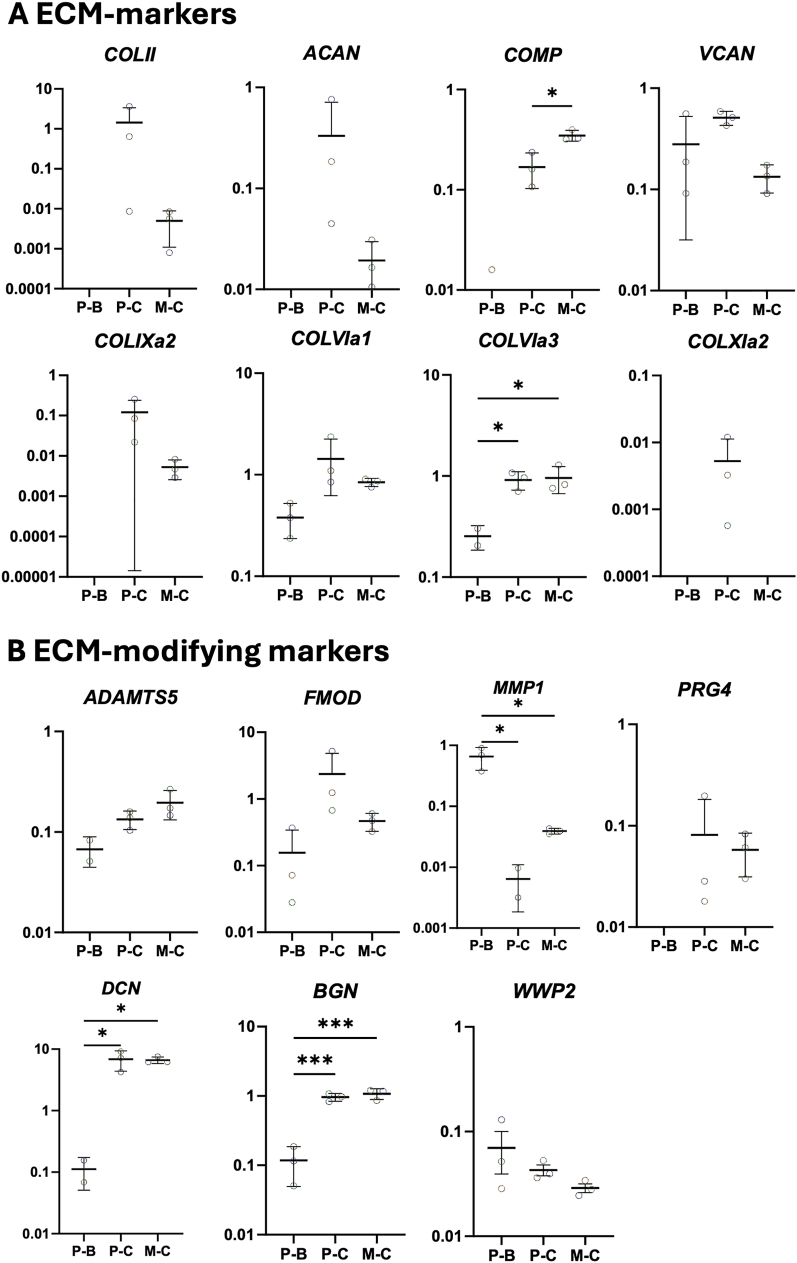
Normalised gene expression of ECM-related genes in pellet and spheroid cultures at 28 days of in vitro culture **A.** ECM-markers. **B** ECM-modifying markers. Dot plots represent the 2ˆ(-ΔCt). Biological replicates are n = 3. Each dot represents one donor. Abbreviations in graphs: P – B (Basic Pellet), P – C (Chondrogenic Pellet), and M – C (Chondrogenic Microwell). ∗p < 0.05; ∗∗p < 0.01; ∗∗∗p < 0.001.

For chondrogenic ECM-related genes (Fig. 5A), a similar expression trend was observed for Collagen type II (*COLII*) and Aggrecan (*ACAN*), with chondrogenic pellets showing higher expression compared to spheroids (*COLII*; 1.43 ± 1.59 vs 0.005 ± 0.003 and *ACAN*; 0.33 ± 0.31 vs 0.02 ± 0.01). However, the observed differences did not reach statistical significance due to donor variability. Interestingly, Cartilage Oligomeric Matrix Protein (*COMP*) expression was significantly 2-fold increased in the chondrogenic microwell condition (0.35 ± 0.04; p < 0.05) compared to the chondrogenic pellet (0.17 ± 0.05). Versican (*VCAN*) followed a similar trend as *COLII* and *ACAN*, with higher expression in pellets compared to spheroids (0.51 ± 0.07 vs 0.13 ± 0.03). In contrast, Collagen type IXa2 (*COLIXa2*) and Collagen type VIa1 expression were elevated in 12-fold and 1.7-fold respectively in the chondrogenic pellets compared to the spheroids (*COLIXa2*; 0.12 ± 0.10 vs 0.01 ± 0.002 and *COLVIa1*; 1.43 ± 0.66 vs 0.84 ± 0.06), while Collagen type VIa3 showed significant increase of the chondrogenic pellet (0.91 ± 0.15; p < 0.05) and spheroid (0.96 ± 0.23; p < 0.05) compared to the basic pellet (0.25 ± 0.05). Collagen type X (COLX)Ia2 (*COLXIa2*) was only expressed in the chondrogenic pellets.

For ECM-modifying genes (Fig. 5B), A-Disintegrin and Metalloproteinase with Thrombospondin Motifs-5 (*ADAMTS5*) expression was upregulated in both chondrogenic conditions, with 1.5-fold higher levels in spheroid cultures (0.20 ± 0.05) compared to the pellets (0.13 ± 0.02), possibly associated with the observed lower expression of *ACAN* and *VCAN*. Fibromodulin (*FMOD*), involved in matrix remodelling, was most highly expressed in chondrogenic pellets in a 15-fold and 5-fold manner, respectively, compared to the basic pellets and spheroids. The Matrix Metalloproteinase 1 levels were significantly higher in the basic pellets (0.66 ± 0.22) compared to the chondrogenic pellets (0.01 ± 0.003; p < 0.05) and spheroids (0.04 ± 0.004; p < 0.05). Decorin (*DCN*) and Biglycan (*BGN*) were significantly increased in the chondrogenic pellets (*DCN*; 6.86 ± 2.03; p < 0.05 and *BGN*; 0.96 ± 0.10; p < 0.0001) and spheroids (*DCN*; 6.64 ± 0.67; p < 0.05 and *BGN*; 1.08 ± 0.16; p < 0.0001) compared to the basic pellets (*DCN*; 0.11 ± 0.04 and *BGN*; 0.12 ± 0.06), but have comparable relative expression between the two chondrogenic culture systems. For other ECM-modifying genes, including Proteoglycan 4 (*PRG4)* and WW Domain Containing E3 Ubiquitin Protein Ligase 2 (*WWP2*), no significant differences were observed between the two culture systems.

**Fig. 6:**
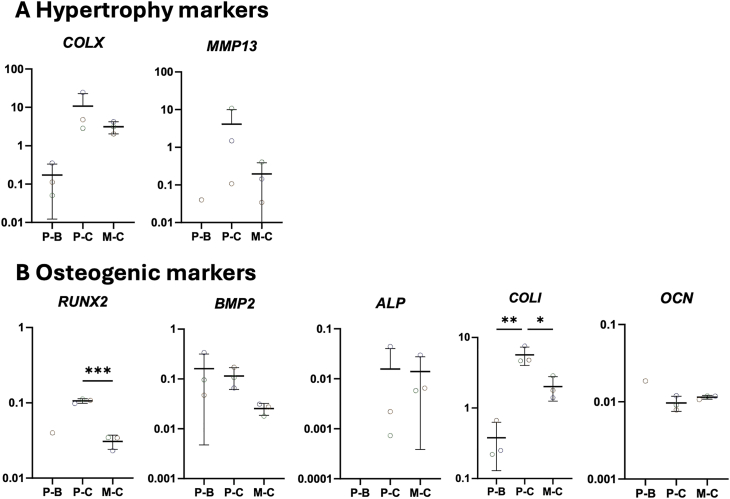
Normalised gene expression of hypertrophic and osteogenic genes in pellet and spheroid cultures at 28 days of in vitro culture **A.** Hypertrophy markers. **B** Osteogenic markers. Biological replicates is n = 3. Each dot represents one donor. Dot plots represent the 2ˆ(-ΔCt). Abbreviations in graphs: P – B (Basic Pellet), P – C (Chondrogenic Pellet), and M – C (Chondrogenic Microwell). ∗p < 0.05; ∗∗p < 0.01; ∗∗∗p < 0.001.

Next, we assessed the expression of hypertrophic markers (Fig. 6A). Chondrogenic pellets exhibited higher expression of hypertrophic markers Collagen type X (*COLX*) (10.73 ± 9.81) and Matrix Metalloproteinase 13 (*MMP13*) (4.14 ± 4.76) compared to spheroids (*COLX*; 3.12 ± 0.89 and *MMP13*; 0.20 ± 0.16), corresponding to a 3.4-fold increase in *COLX* and a 20.7-fold increase in *MMP13*. Lastly, we investigated the expression of osteogenic markers in both culture systems (Fig. 6B). Runt-Related Transcription Factor 2 (*RUNX2*) expression levels were significantly reduced in the microwell culture condition (0.03 ± 0.01; p < 0.0001) compared to the chondrogenic pellets (0.11 ± 0.01), with Bone Morphogenic Protein 2 (*BMP2*) also showing a noticeable reduction of 3.6-fold. Alkaline Phosphatase (*ALP*) expression remained comparable between the two systems (0.02 ± 0.02 vs 0.01 ± 0.01). Collagen type I (*COLI*) levels were also significantly decreased in the spheroid culture (2.01 ± 0.62; p < 0.05) compared to the chondrogenic pellets (5.67 ± 1.35), whereas Osteocalcin (*OCN*) expression showed no differences between the chondrogenic pellet (0.01 ± 0.002) and spheroid cultures (0.01 ± 0.001).

### 3.3 Histological findings

**Fig. 7:**
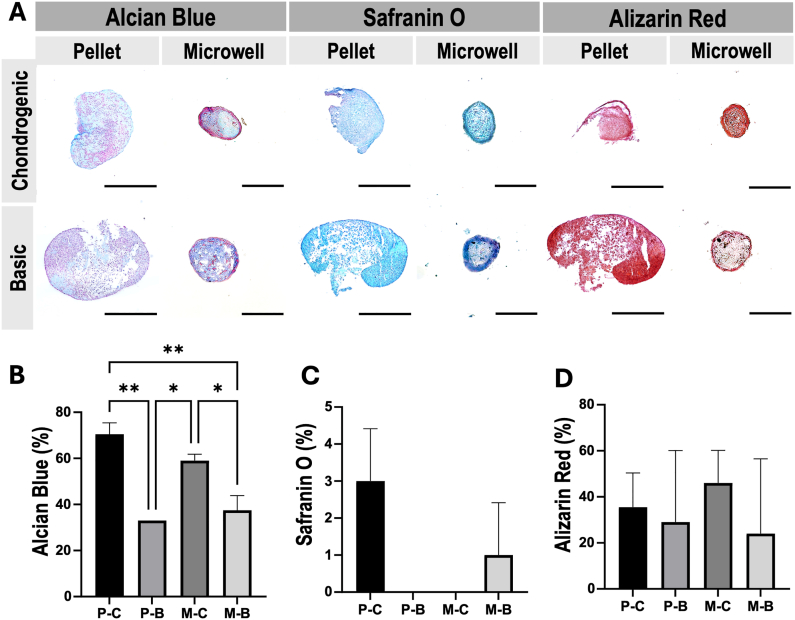
**A.** Alcian Blue, Safranin O, and Alizarin Red staining in chondrogenic pellet and spheroids, as well as pellets cultured in basal conditions at 28 days of in vitro culture. Semi-quantitative analysis of positively stained areas, performed by ImageJ software and reported as % of the total area, for **B.** Alcian Blue, **C.** Safranin O, and **D.** Alizarin Red. The scale bar in the pellet aggregates represents 500 μM, while the microwell-formed spheroids represent 100 μM. Abbreviations in graphs: P–C (Chondrogenic Pellet), P – B (Basic Pellet), M – C (Chondrogenic Microwell), and M – B (Basic Microwell). ∗p < 0.05; ∗∗p < 0.01. (For interpretation of the references to color in this figure legend, the reader is referred to the Web version of this article.)

**Fig. 8:**
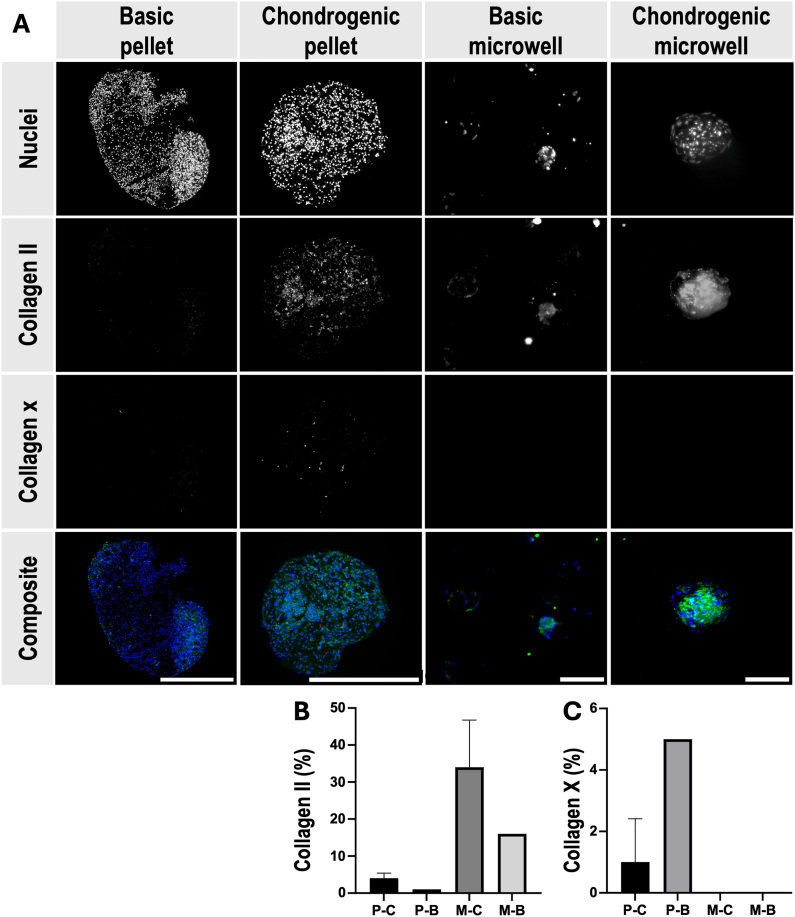
IHC of collagen type II and collagen type X of chondrogenic pellet and spheroids, as well as pellets cultured in basal conditions at 28 days of in vitro culture. Semi-quantitative analysis of positively stained areas, performed by ImageJ software and reported as % of the total area, for **B.** collagen type II and **C.** collagen type X. The scale bar in the pellet aggregates represents 500 μM, while the microwell-formed spheroids represent 100 μM. Abbreviations in graphs: P – C (Chondrogenic Pellet), P – B (Basic Pellet), M – C (Chondrogenic Spheroid), and M – B (Basic Microwell).

To further evaluate the chondrogenic ECM composition of hBMSC-derived spheroids and pellets, we performed histological staining and IHC after 28 days of in vitro culture (Fig. 7, Fig. 8). Alcian blue staining revealed an ECM richer in sulphated Glycosaminoglycans (GAGs) in the chondrogenic pellet condition compared to spheroids (Fig. 7A and B). Safranin O staining, slightly visible as red in the periphery of the chondrogenic pellets, indicated proteoglycan deposition, which was not observed in spheroids (Fig. 7A–C). Albeit modest, these findings suggest an advantage of pellet cultures over spheroids in chondrogenic ECM deposition.

Alizarin red staining revealed calcium deposits in both chondrogenic and basic aggregates, with less mineralisation observed in spheroids (Fig. 7A–D). This supports the qPCR findings that hypertrophy and osteogenesis were less present in spheroids compared to pellets.

Immunohistochemical analysis further highlighted differences in matrix composition. COL-II was detected in both chondrogenic pellet and spheroid cultures. However, COL-II distribution was more diffuse in the pellet culture, while in spheroids, it appeared more concentrated (Fig. 8A and B). In contrast, COL-X, a marker of hypertrophy, was exclusively present in the pellet culture, further supporting the observations of reduced hypertrophy in the microwell spheroid system (Fig. 8A–C).

## 4 Discussion

MSCs have emerged as a promising cell source for cartilage regeneration due to their ability to differentiate into chondrocytes [3]. However, these cells tend to exhibit hypertrophy during chondrogenesis, thereby posing a significant challenge to their use for clinical applications. This study evaluated two 3D culture systems, i.e., microwell and pellet culture, concerning their utility in supporting the chondrogenic differentiation of MSCs while eradicating the occurrence of hypertrophy. All comparisons between pellet and spheroid cultures were conducted using optimised seeding densities, selected based on preliminary experiments to ensure consistent and representative 3D-structure formation. Our results showed a more effective chondrogenesis in pellet cultures. However, spheroid cultures showed decreased expression of hypertrophic markers. Other research groups have demonstrated that the dimensions of cellular aggregates in culture influence their chondrogenic potential [3,14]. Interestingly, those observations are often conflicting, with both larger and smaller dimensions of the aggregates associated with improved chondrogenesis and decreased hypertrophy [3,14]. This emphasises the need to study these processes in closer detail for in vitro models to investigate cartilage tissue.

Our IHC results revealed the expression of COL-II in the microwell-formed spheroids, whereas both COL-II and COL-X were present in the pellet culture. This may indicate the presence of a mixed population of chondrocitic cells in the pellets, with some cells in an early, more proliferative stage of chondrogenesis and others transitioning towards hypertrophy. Histological analysis revealed ECM content rich in GAGs and peripheral proteoglycan deposition in the chondrogenic pellets, with reduced deposition towards the centre. This is likely due to the spatial heterogeneity of hBMSCs differentiation in pellet culture, where cells in the outer layer have more efficient chondrogenesis [3]. Microwell-formed spheroids had more uniform ECM formation and less mineralisation, which likely results from the small size of the cellular aggregates, which might improve gas and nutrient diffusion [15].

Further evidence supporting these observations was provided by gene expression analysis. The higher expression of cartilage-specific genes, including *COL II*, *COLIXa2*, and *COL XIa2*, *ACAN*, and *VCAN* in chondrogenic pellets, indicates a more robust chondrogenesis. Elevated *SOX5*, *SOX6*, and *SOX9* expression in the chondrogenic pellets also supported the cartilage-specific gene expression observed. *SOX9* acts independently but collaborates with *SOX5* and *SOX6* to transactivate target genes like *COLII*, *COL IXa2*, and *COLXIa2*, as well as *ACAN* [16,17]. The higher expression of chondrogenic markers observed in the pellet culture compared to the spheroid culture could be attributed to the higher cell density and enhanced cell-cell interactions, which are known to more closely mimic mesenchymal condensation during chondrogenesis [18,19]. Similar expression of pericellular matrix genes in both chondrogenic pellets and microwell-formed spheroids, such as *COLVIa1* and *COLVIa3*, suggests active ECM stabilisation [20,21].

Minimal or absent expression of these chondrogenic genes in the basic pellets indicates a lack of transcriptional activity, confirming that chondrogenesis was successfully induced solely in the chondrogenic groups. In addition, the absence of *PTH1R* expression, a gene associated with chondrogenic differentiation, in the basic pellets is consistent with the observed lack of transcriptional activity [8]. *DCN* and *BGN* were markedly elevated in chondrogenic pellets and microwell cultures compared to the basal pellets, reflecting the limited chondrogenesis in the latter. *DCN* serves as a physical linker between *ACAN* and *COLII* through retention and thereby slows down *ACAN* loss, while *BGN* modulates signalling during chondrogenesis and controls the ECM structure [[22], [23], [24]]. Although relative *DCN* and *BGN* expression levels were comparable between chondrogenic pellets and spheroids, it is important to note that *DCN* upregulation does not directly drive *ACAN* expression. In spheroids, the lower *ACAN* levels may indicate a compensatory shift toward ECM stabilisation that prioritises network integrity and pericellular matrix formation over *ACAN* expression. *BGN* expression in the spheroids, along with the modest chondrogenic marker expression, suggests that the spheroids retain some chondrogenic activity but may reflect incomplete chondrogenic maturation or a shift toward ECM stabilisation.

*COMP*, which helps assemble and stabilise collagen networks within cartilage [25], was significantly higher in the spheroids, despite overall lower chondrogenesis. This may reflect a specific role in matrix stabilisation and network organisation rather than the extent of chondrogenic differentiation, potentially driven by the compact spheroid microenvironment and associated mechanical cues.

Hypertrophic markers were lower or comparable in the chondrogenic spheroids when compared to pellets; reduced *COLX* expression was found in the spheroids. The lower expression of *COLX*, a critical regulator of *COLII* levels [26], in the chondrogenic spheroids provides a plausible explanation for the simultaneous reduction of *COLII*, linking hypertrophic suppression with altered cartilage matrix formation in the microwells. This gene regulation is consistent with the expression patterns observed for the overall chondrogenic markers investigated. The elevated *COLX* expression in the chondrogenic pellet culture may result from higher cell density and cell-cell interactions, which more closely recapitulate mesenchymal condensation during chondrogenesis [19]. Indeed, these pellets showed increased hypertrophic differentiation, potentially driven by the significant increase of *RUNX2*, which may underlie the observed increases in CO*LI*, *COLX,* and *MMP13* expression [27]. In contrast, the *ADAMTS5* expression in the spheroids suggests greater ECM remodelling, as *ADAMTS5* plays a role in collagen and *ACAN* degradation [28,29], which is consistent with the observed lower expression of *ACAN* and *COLII* in the chondrogenic spheroids.

Interestingly, the hypoxia markers *HIF1a* and *EPAS1* were expressed at higher levels in the basic culture conditions compared to hBMSCs undergoing chondrogenesis, despite all cultures being maintained under normoxic conditions. This likely reflects the undifferentiated state of the cells [30], the influence of FBS supplementation in the basal group, and their metabolic state [31] rather than indicating enhanced chondrogenic potential.

*HIF1α*, which is known to induce the expression of *SOX9*, *COLII*, and *ACAN* [32,33], was the lowest in the chondrogenic microwells, although this difference did not reach statistical significance. It should be noted that all experiments were conducted in hyperoxic conditions (20% oxygen), under which *HIF1α* is known to undergo degradation and reduced transcriptional activity [34]. Given the smaller size of the microwell aggregates and their improved oxygen and nutrient diffusion [15]. These cells may not experience the same degree of hypoxia as in pellet aggregates. While oxygen gradients and associated activation of hypoxia-responsive pathways have been reported to enhance chondrogenesis of MSCs [[35], [36], [37], [38]], the absence of direct oxygen measurements in this study precludes any mechanistic conclusions regarding their role in our system. Similarly, *EPAS1* expression was the lowest in chondrogenic pellets, despite *EPAS1* being reported to promote key chondrogenic markers [39]. The divergent regulation of *HIF1α* and *EPAS1* suggests that hypoxia-related signalling is unlikely to be a driver of the observed differences between the two culture systems.

Our study, albeit revealing, features a few limitations that should be considered. There is a lack of spheroid culture without chondrogenic stimulation that could provide insides on the baseline behaviour of this system. Additionally, we used hBMSCs at passage 5 to ensure sufficient cell numbers and stable phenotypic characteristics. However, cells at earlier passages might feature higher stemness and be more prone to chondrogenic differentiation under the given stimuli. Therefore, our findings may not fully capture the variability across all passages. As mentioned earlier, our investigations were performed in hyperoxic conditions. The role of hypoxia as an underlying mechanism of chondrogenesis warrants further investigation. Although the 28-day endpoint employed by us allowed for robust chondrogenesis and ECM production, earlier timepoints could provide valuable insights into initial gene expression (e.g., *COLII*, *SOX9*) and initial ECM deposition. Finally, we observed donor-to-donor variability in gene expression, reflecting inherent biological heterogeneity. While this variability can affect the statistical significance and the interpretation of the results should be done with caution, including multiple donors strengthens the relevance of our findings across different backgrounds.

In conclusion, **t**his study highlights the complexities of MSCs’ chondrogenesis and its regulation across different culture systems, highlighting the roles of chondrogenesis, hypertrophy, and transcription factors. Spheroids formed by utilizing microwell culture systems offer significant advantages in reducing hypertrophy and matrix organisation, highlighting their potential in settings where hypertrophy suppression, reproducibility, or matrix organisation are a priority. Unexpectedly, this system did not support the expression of chondrogenic differentiation markers when compared to 3D pellet cultures. It may be relevant to further explore hypoxic conditions and the use of chondrogenic bioactive compounds to harvest the best chondrogenic features on this advanced, 3D culture system. The complementary strengths of microwell platforms suggest that they may be better suited for alternative translational applications requiring tighter regulation of tissue maturation or scalable microtissue production.

Overall, our findings underscore that the choice of culture system should be guided by the specific biological and translational objectives, with both in vitro culture platforms offering distinct and complementary strengths, which may help future studies by aligning experimental models with specific chondrogenic objectives.

## Ethics statement

Bone marrow samples were harvested from three patients’ iliac crest sites at Maastricht University Medical Center, MUMC+, Maastricht, the Netherlands, after written informed donor consent. Specifically, the three specimens were collected as a liquid biopsy from the acetabulum of patients admitted to the clinic for coxarthrosis (n = 2) or spine surgery (n = 1). Patients include a female and two male donors with ages in the range of 64–68 years old. Ethical approval was obtained from the local ethical committee of the MUMC+, with the approval number METC 15-4-274. All procedures were carried out following the latest amendment of the Declaration of Helsinki.

## Author contributions

All authors have made substantial contributions to the conception and design of the study, acquisition and analysis of data, drafting or revising the manuscript, and have approved the final version for submission. In detail, DMAJS: Investigation, data analysis, visualisation, writing-original draft. SG: Visualisation, writing-review and editing. MvG: Conceptualisation, Writing-review and editing. SV: Conceptualisation, methodology, writing-review and editing. ERB: Conceptualisation, methodology, writing-review and editing, funding acquisition.

**Corresponding author responsible for the integrity of the work as a whole:** Prof. Dr. E. Rosado Balmayor (erosadobalma@ukaachen.de).

## Declaration of generative AI in scientific writing

The authors declare that no Generative AI was used in the creation of this manuscript.

## Role of funding source

This work was partially funded by the Province of Limburg, Limburg Invests in its Knowledge Economy (LINK) to MvG and the Gravitation Program of the Netherlands Organization for Scientific Research NWO (MDR, grant 024.003.013) to SV. Open access funding provided by the Open Access Publishing Fund of RWTH Aachen University.

## Competing interests

SG is founder and shareholder of the company 300MICRONS GmbH. The authors have no other conflict of interest to declare.
