# Supplementary material for: Understanding the use of spheroids and pellets in the chondrogenic differentiation of human stem cells
Source: Osteoarthr Cartil Open. 2026 Mar 14;8(2):100779. doi: 10.1016/j.ocarto.2026.100779 (PMC13051975; doi:10.1016/j.ocarto.2026.100779)
Supplement: Multimedia component 1 [file mmc1.docx]

**Supplementary Table 1**: Reference Target Stability (geNORM M-values) of the three reference genes used: *CPSF6*, *GAPDH*, and *TBP*. The table is exported from qBASE+ software. qPCR was performed in two sets of technical replicates. **A.** First set of replicates. **B.** Second set of replicates.

**A.**

| **Reference Target** | **M-value** |
| --- | --- |
| *CPSF6* | 0.863 |
| *GAPDH* | 0.991 |
| *TBP* | 0.663 |
| Average | 0.839 |

**B.**

| **Reference Target** | **M-value** |
| --- | --- |
| *CPSF6* | 2.013 |
| *GAPDH* | 2.826 |
| *BTBP* | 2.031 |
| Average | 2.290 |
